# Establishment and evaluation of transgenic models of Alzheimer's disease with senescence accelerated background

**DOI:** 10.1186/1750-1326-7-S1-S34

**Published:** 2012-02-07

**Authors:** Wenjuan Zhao, KengHoe Lok, Hong Zhao, Anyang Sun, Ming Yin

**Affiliations:** 1 Laboratory of Neuropharmacology, School of Pharmacy, Shanghai Jiao Tong University, 200240, Shanghai, China

## Background

Although transgenic animal models of Alzheimer’s disease (AD) have proven to be relatively faithful models for neuropathological changes, none completely recapitulate the disease process. We aimed to establish new AD transgenic models with senescence accelerated mouse prone 8 (SAMP8) background.

## Method

In the experiments, APP/PS1 and tau^P301L^ transgenic mice of C57BL/6J background were interbred with SAMP8 mice for five generations. Gene identification of filial generation was tested by PCR. The grading score system developed by Takeda T *et al* was used for evaluation of the degree of senescence in mice. The age-related decline of learning and memory ability was analyzed using passive avoidance shuttle box and Morris water maze tasks. Immunofluorescence study was performed to ascertain the presence of β-amyloid (Aβ) and tau pathology in AD mouse models by using β-amyloid antibody and specific phosphorylated tau antibody at Ser404.

## Results

Both APP/PS1 and tau^P301L^ transgenic mice in SAMP8 background exhibited accelerated aging with a high grading score. The transgenic mice with SAMP8 background also exhibited age-dependent cognitive impairments, compared with transgenic mice with C57BL/6J background or wild mice with SAMP8 background at the same age. In shuttle box avoidance task, tau^P301L^ transgenic mice with SAMP8 background had obvious memory deficit very early in life, approximately at the age of 6-month old. Probe trial in Morris water maze test showed a marked deficit in the APP/PS1 transgenic mice with SAMP8 background, which spent less time at platform quadrant, compared with APP/PS1 transgenic mice with C57BL/6J background or wild mice with SAMP8 background at the same age of 9-month old. In immunohistochemistry studies, SAMP8 mice showed sporadic Aβ deposition in the hippocampus, cortex and thalamus from as early as 6-month old, and increase in number and extent with age. Significant elevation of Aβ deposition in the whole brain, even extent to the cerebellum and spinal cord of 6--month-old APP/PS1 transgenic mice with SAMP8 background could be seen. We also found that obvious immunofluorescent staining of phosphorylates tau at Ser404 (PHF-tau) in the hippocampus, cortex and thalamus 9-month-old tau^P301L^ transgenic with SAMP8 background. However, no obvious phosphorylated tau were found in wild SAMP8 mice or tau^P301L^ transgenic C57BL/6J mice even at the age of 9 months, when compared with wild C57BL/6J mice.

## Conclusion

The characterized neuropathological alterations and cognitive impairment in AD can be reproduced genetically early in transgenic mice over-expressing APP/PS1 or tau^P301L^ genes with senescence accelerated background. These novel mouse models for AD will help to clarify the pathogenic mechanism and allow assessment of the effects of new treatment strategies.

